# Estimating uncertainty in geospatial modelling at multiple spatial resolutions: the pattern of delivery via caesarean section in Tanzania

**DOI:** 10.1136/bmjgh-2019-002092

**Published:** 2020-02-10

**Authors:** Corrine Warren Ruktanonchai, Jeremiah J Nieves, Nick W Ruktanonchai, Kristine Nilsen, Jessica E Steele, Zoe Matthews, Andrew J Tatem

**Affiliations:** 1 School of Geography & Environmental Science, University of Southampton, Southampton, UK; 2 Department of Social Statistics & Demography, University of Southampton, Southampton, UK

**Keywords:** maternal health, geographic information systems, epidemiology

## Abstract

Visualising maternal and newborn health (MNH) outcomes at fine spatial resolutions is crucial to ensuring the most vulnerable women and children are not left behind in improving health. Disaggregated data on life-saving MNH interventions remain difficult to obtain, however, necessitating the use of Bayesian geostatistical models to map outcomes at small geographical areas. While these methods have improved model parameter estimates and precision among spatially correlated health outcomes and allowed for the quantification of uncertainty, few studies have examined the trade-off between higher spatial resolution modelling and how associated uncertainty propagates. Here, we explored the trade-off between model outcomes and associated uncertainty at increasing spatial resolutions by quantifying the posterior distribution of delivery via caesarean section (c-section) in Tanzania. Overall, in modelling delivery via c-section at multiple spatial resolutions, we demonstrated poverty to be negatively correlated across spatial resolutions, suggesting important disparities in obtaining life-saving obstetric surgery persist across sociodemographic factors. Lastly, we found that while uncertainty increased with higher spatial resolution input, model precision was best approximated at the highest spatial resolution, suggesting an important policy trade-off between identifying concealed spatial heterogeneities in health indicators.

**Key questions**

**What is already known?**

- Estimating maternal and newborn health outcomes at small geographical areas is increasingly important in identifying hidden pockets of health inequalities. The use of Bayesian geostatistical models has allowed for the quantification of associated uncertainty with these modelled estimates.

**What are the new findings?**

- The trade-off between increasing spatial resolution in model inputs/outcomes and associated uncertainty has not been explored, particularly among maternal and newborn health outcomes.

**What do the new findings imply?**

- While uncertainty in model outcomes increases with increasing spatial resolution, model precision was best approximated at the finest spatial resolution for prevalence of delivery via caesarean section in Tanzania. These findings imply an important trade-off between identifying concealed spatial heterogeneities and accuracy of estimates, which should be communicated in policy relevant settings.

## Introduction

Achieving the Sustainable Development Goal aims laid out in 2015 necessitates measurement of health outcomes at small geographical areas to ensure ‘no one left behind’.^1^ With recent advancements in the collection and distribution of geo-located household surveys, such as those collected via the Demographic and Health Survey (DHS) programme (www.dhsprogram.com), researchers are increasingly using methods such as small area estimation and geostatistical additive models (GAMs) to generate high spatial resolution maps of health and development indicators.^2–4^ Such subnational, high-resolution estimates have become useful tools for researchers and policy makers alike in uncovering hidden health inequities that would otherwise be masked by aggregate or national-level health indicators, enabling targeted interventions in settings with limited resources.^1 5–8^

Visualising health outcomes and associated uncertainty at high spatial resolutions has distinct policy relevance among maternal and newborn health (MNH) outcomes,^9^ as maternal and neonatal mortality both vary geographically and occur relatively rarely. Furthermore, the data associated with maternal mortality are subject to limitations, misclassification and bias,^10 11^ particularly within more rural areas of sub-Saharan Africa where many deaths do not occur at hospitals and may go unrecorded.^12^ As with maternal mortality, data on life-saving MNH interventions such as antenatal care, skilled birth attendance and delivery via caesarean section (c-section) can be widely obtained at aggregate levels but remain difficult to measure at subnational levels, especially in the most rural and vulnerable areas of the world. While some work has been done modelling key MNH interventions at subnational scales such as maternal health services, exclusive breastfeeding, childhood vaccinations and health systems performances,^5 6 13–15^ other vital life-saving interventions that occur less frequently, such as delivery via c-section, have not been modelled previously at high spatial resolutions.

With advancements in computational resources and data availability over recent decades, researchers across disciplines have begun employing Bayesian GAM to map disease and quantify uncertainty in posterior model outcomes, particularly using hierarchical clustered data such as from the DHS.^3 4 16–18^ The application of these methods is increasingly pertinent, as access to healthcare services is heterogeneously distributed across landscapes, requiring high-resolution spatial data and modelling techniques to identify the most vulnerable populations. However, these methods and associated spatial data carry limitations and bias, manifesting in uncertainty that should be adequately quantified and communicated to decision makers and non-academic audiences for optimum policy impact.^19^ While the use of such GAMs to predict high-resolution health outcomes has improved model parameter estimates and precision among spatially correlated and rare adverse health outcomes^3 20^ and allowed for this quantification of uncertainty,^21^ no studies have examined the trade-off between predicting health outcomes at higher spatial resolutions and visualising the spatial distribution of associated uncertainty.^22^

Here, we estimate prevalence of delivery via c-section in Tanzania, using input covariates at varying levels of spatial coarseness within a Bayesian geostatistical model framework. With these models, we investigate how uncertainty varies with spatial resolution, and how this changing uncertainty can be better visualised and communicated. Specifically, we explore the trade-off between model estimates and associated uncertainty at increasing spatial resolutions through exploration of the posterior distribution of modelled delivery via c-section at multiple spatial resolutions.

## Methods

### DHS data

**Figure 1:**
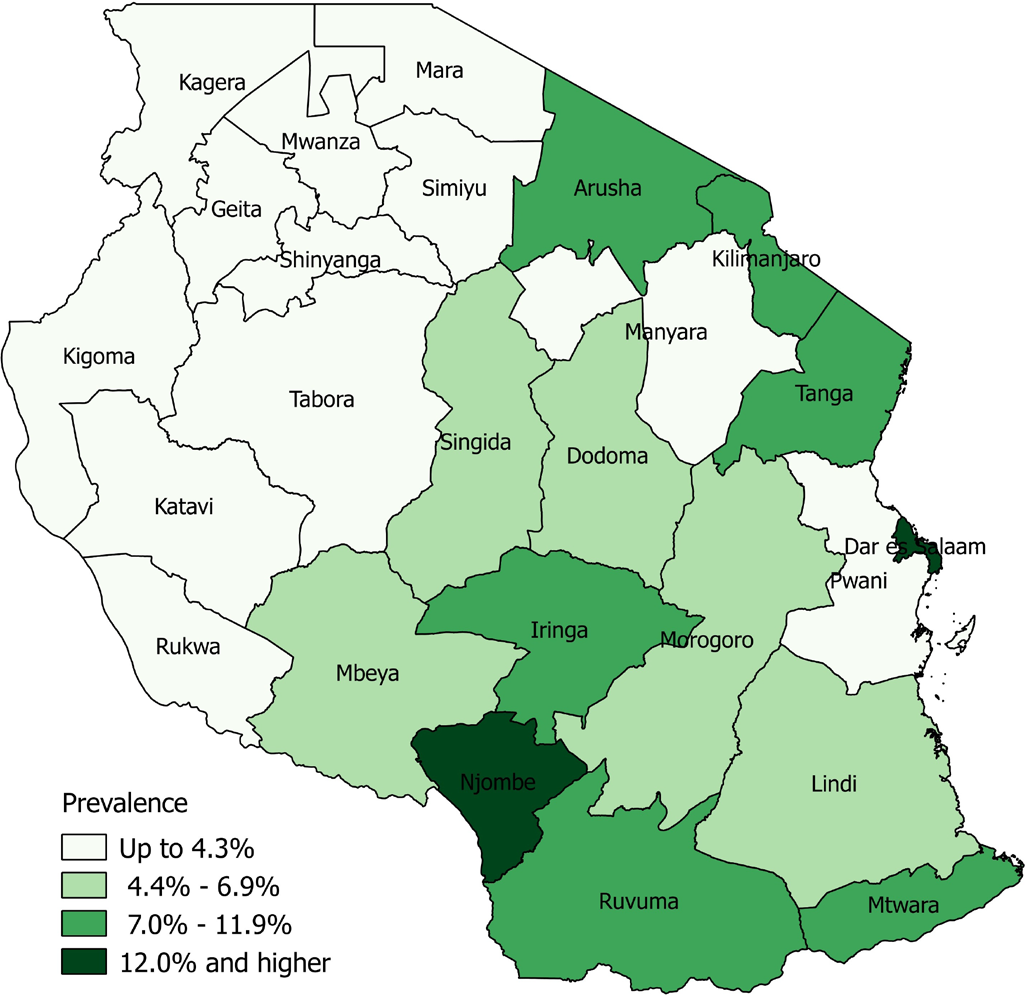
Delivery by caesarean section at the administrative 1 level using DHS data, Tanzania, 2015.

We compiled DHS data from Tanzania for 2015^23^ using SAS V.9.4 software^24^ and restricted the sample to women with a birth in the preceding 5 years (n=7050 women) with corresponding spatial data, as provided by DHS cluster locations. Briefly, the DHS provides global positioning system (GPS) coordinates for clusters of aggregated household survey data in order to facilitate spatial analyses while also maintaining participant confidentiality. These coordinates are displaced up to 2 km in urban areas and 5 km in rural areas, with up to 1% of points displaced up to 10 km in rural areas.^25^ Using these geo-located cluster locations, spatial inference occurs at a higher spatial resolution than the geographic region in which the survey is designed to be representative of. This hierarchical sample design therefore necessitates the use of geostatistical models to make inferences at spatial resolutions finer than the DHS region level.^3^ In these analyses, our binary outcome of interest was defined as the number of women who underwent any delivery via c-section for a birth within the preceding 5 years (regardless of whether it was the most recent), as compared with women who had not experienced delivery via c-section for any preceding birth. To maintain survey representativeness, this was calculated at the DHS level 1 resolution, representing the 30 administrative I regions of Tanzania (figure 1).

### Covariate data

**Figure 2:**
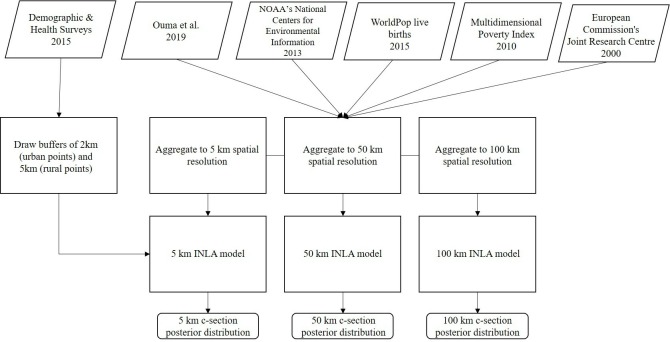
Study analysis flow chart. INLA, Integrated Nested Laplace Approximation; NOAA, National Oceanic and Atmospheric Administration.

In addition to demographic data gathered through the DHS, we also compiled environmental geospatial covariate data that we extracted to DHS cluster locations (figure 2). Because these locations are displaced, we averaged geospatial covariates to 2 km and 5 km buffers for urban and rural locations, respectively. While up to 1% of coordinates in rural areas are displaced within a 10 km radius, the addition of buffers at the 10 km level has been shown to impact very few rural coordinates,^25^ while unnecessarily introducing bias in environmental covariates, thereby justifying use of a 5 km buffer in rural areas. First, we gathered data from the European Commission’s Joint Research Centre on accessibility to major cities for the year 2000, representing travel time to the nearest city exceeding a population of 50 000 using land (ie, roads) or water-based travel (ie, rivers and lakes).^26^ Next, we included data on annual night light intensity for the year 2013 (an indicator of urbanicity), as generated by the National Oceanic and Atmospheric Administration’s National Centers for Environmental Information.^27^ We also included live births for the year 2015 at the 1 km resolution, as well as Multidimensional Poverty Index estimates for the year 2010, as obtained via the WorldPop Project (www.worldpop.org.uk) and outlined by Tatem *et al*.^28 29^ Lastly, we included travel time to the nearest public hospital, as outlined by Ouma *et al*,^30^ calculated through a cost–distance algorithm incorporating a travel impedance surface by assigning travel speed to road networks.

These covariates were chosen as previous studies have shown them to be predictive of MNH outcomes and risks,^6 31–34^ representing a suite of geospatial covariates with robust predictive power to examine how uncertainty changes as a function of spatial resolution. Notably, we chose to include only geospatial covariates in this model as we could vary the spatial resolution of these variables, thereby addressing our research objectives. Specifically, these datasets were compiled at the 1 km spatial resolution and subsequently averaged at the 5 km, 50 km and 100 km resolutions to facilitate projecting the fitted model onto gridded surfaces at these levels. These surfaces represent a theoretical exploration of the trade-off between increasing gridded spatial resolution and modelled estimates and were chosen to clearly illustrate the difference in the practical range of estimates, as shown in online supplementary figure S2.

10.1136/bmjgh-2019-002092.supp1 Supplementary data

### Model framework

To explore how uncertainty in posterior modelled c-section delivery estimates varied at multiple spatial resolutions, we employed a Bayesian hierarchical model framework with input covariates at varying spatial coarseness. These models have been used extensively with DHS and other household survey data,^1 2 35–38^ as they are able to robustly account for the multistage sampling efforts employed during the data collection process, resulting in hierarchically structured data provided through the DHS. Here, our model accounts for the nested structure of DHS data by allowing for variation in the *n* ^th^ region among individual respondents, as outlined below. These models were fit independently of each other, resulting in three models with input covariates and modelled outcomes at the 5 km, 50 km and 100 km spatial scales. To predict c-section delivery at a continuous spatial resolution, we implemented these models via stochastic partial differential equation (SPDE) spatial regression approach, implemented using the Integrated Nested Laplace Approximation (INLA) technique within the R-INLA package.^39^ This approach was suitable for this analysis as these spatial processes are generally well captured by a Gaussian field with Matérn correlation.^40^ These models have similarly been used in previous research combining DHS data and geospatial covariates to predict high-resolution childhood vaccination coverage by disaggregating areal surveillance data.^1 2 34^ We employed a similar modelling framework, generally defined as

$$Y_{i}∼Binomial\left(N_{i},p_{i}\right),i=1,\ldots ,n_{A},n_{A}+1,n_{A}+n_{p}$$

where $n_{A}$ represents subnational DHS regions within Tanzania to maintain survey representation; $Y_{i}$ represents the number of women delivering via c-section within area, $A_{i}$; $p_{i}$ represents the probability of a woman delivering via c-section over grid points $n_{p}$; and $N_{i}$ represent the number of women surveyed within area, $A_{i}$.

In this framework, the areal units and observation grid points are linked using the following equations

$$logit(p_{i})={\tilde{x}}_{i}^{\prime }\beta +|A_{i}{|}^{-1}\int n(s)ds+\phi_{i},i=1,...,n_{A}$$

$$logit(pi)=x_{i}^{\prime }\beta +\eta \left(s_{i}\right)+\phi_{A_{i}},i=n_{A}+1,\ldots ,n_{A}+n_{p}$$

where $x_{i}$ and ${\tilde{x}}_{i}$ represent covariates for the *i^th^* area and grid point, respectively. This framework provides a statistical link between the areal data and high-resolution spatial covariates and random effects, allowing for models at two spatial levels. Further details on the model framework are outlined in Utazi *et al*.^2^ See refs 2 34 40 for more detailed information on similar approaches implementing an SPDE approach of Bayesian hierarchical models via R-INLA using DHS data.

### Patient and public involvement

Patients or the public were not involved in the design, conduct, reporting or dissemination of our research. The data used in these analyses were obtained from the DHS programme, which makes global health and demographic data confidentially and freely available to researchers across the world. More information on how the DHS programme conducts the Informed Consent process can be found at https://dhsprogram.com.

## Results

Table 1 shows posterior marginal effects for the fixed effects within the 5 km, 50 km and 100 km models as well as model hyperparameters. Fixed effects estimates with upper and lower 95% credible intervals (CIs) that do not cross 1 are considered significant. Overall, we found that modelled c-section prevalence negatively correlated strongly with poverty and slightly with night-time lights across all spatial scales, as shown in online supplementary figure S1. Of note, night-time lights were not significant within the model but presented wide CIs for marginal effects, as seen in table 1. While poverty was not significant at the 5 km scale, it was significant at the 50 km and 100 km scales and showed a consistent pattern at the 5 km scale with other spatial resolutions (online supplementary figure S1). Conversely, these estimates were strongly positively associated with travel time to the nearest hospital across scales, although this was not significant within the model.

**Table 1:** Marginal effects of the fixed effects and hyperparameters of the posterior c-section models at 5 km, 50 km and 100 km

Parameter	5 km			50 km			100 km		
	Mean	Lower 95% CI	Upper 95% CI	Mean	Lower 95% CI	Upper 95% CI	Mean	Lower 95% CI	Upper 95% CI
Accessibility to cities	1.0001	0.9975	1.0027	0.9999	0.9975	1.0022	0.9999	0.9975	1.0022
Night-time lights	0.9603	0.0522	19.7189	0.666	0.0308	14.7623	0.6574	0.0298	14.9301
Live births	1.0471	0.8645	1.2873	0.9915	0.8313	1.1913	0.9938	0.8313	1.197
Poverty	0.0271	0.0005	2.1231	0.0071	0.0001	0.4548	0.0071	0.0001	0.4682
Travel to nearest hospital	1.0046	0.9946	1.0143	1.0062	0.9965	1.0163	1.0063	0.9964	1.0164

Hyperparameters									
Θ_1_	Mean	SD	95% CI	Mean	SD	95% CI	Mean	SD	95% CI
	−0.9259	0.6329	(−2.1791 to 0.3104)	0.229	0.942	(−1.636 to 2.071)	0.213	0.945	(−1.646 to 2.070)
Θ_2_	0.1348	0.4988	(−0.8380 to 1.1226)	0.041	0.724	(−1.376 to 1.472)	0.052	0.718	(−1.352 to 1.474)
Precision	1816.2045	1813.6598	(121.78 to 6626.24)	5.290	5.794	(0.647 to 20.476)	5.814	6.901	(0.648 to 23.539)
λ	0.5020	0.2699	(0.0497 to 0.9491)	0.354	0.243	(0.031 to 0.876)	0.354	0.243	(0.030 to 0.876)
DIC	155.67			152.4			152.61		
P_D_	17.65			17.70			17.81		
Marginal likelihood	−118.52			−120.84			−120.89		

DIC, deviance information criterion.

The large precision estimate for the 5 km model as shown in table 1 suggests the spatial process was estimated well with a Gaussian field, while smaller estimates among the 50 km and 100 km models suggest this was not the case. Regardless, the deviance information criterion (DIC) estimates for the latter two models were slightly improved over the 5 km model (table 1). Briefly, these DIC estimates represent a measure of model comparison, trading off between model complexity and model goodness of fit and performing well among Bayesian models in particular.^41^ Smaller DIC values represent models with better fit, given model complexity, suggesting these models perform better as compared with the other models.

**Figure 3:**
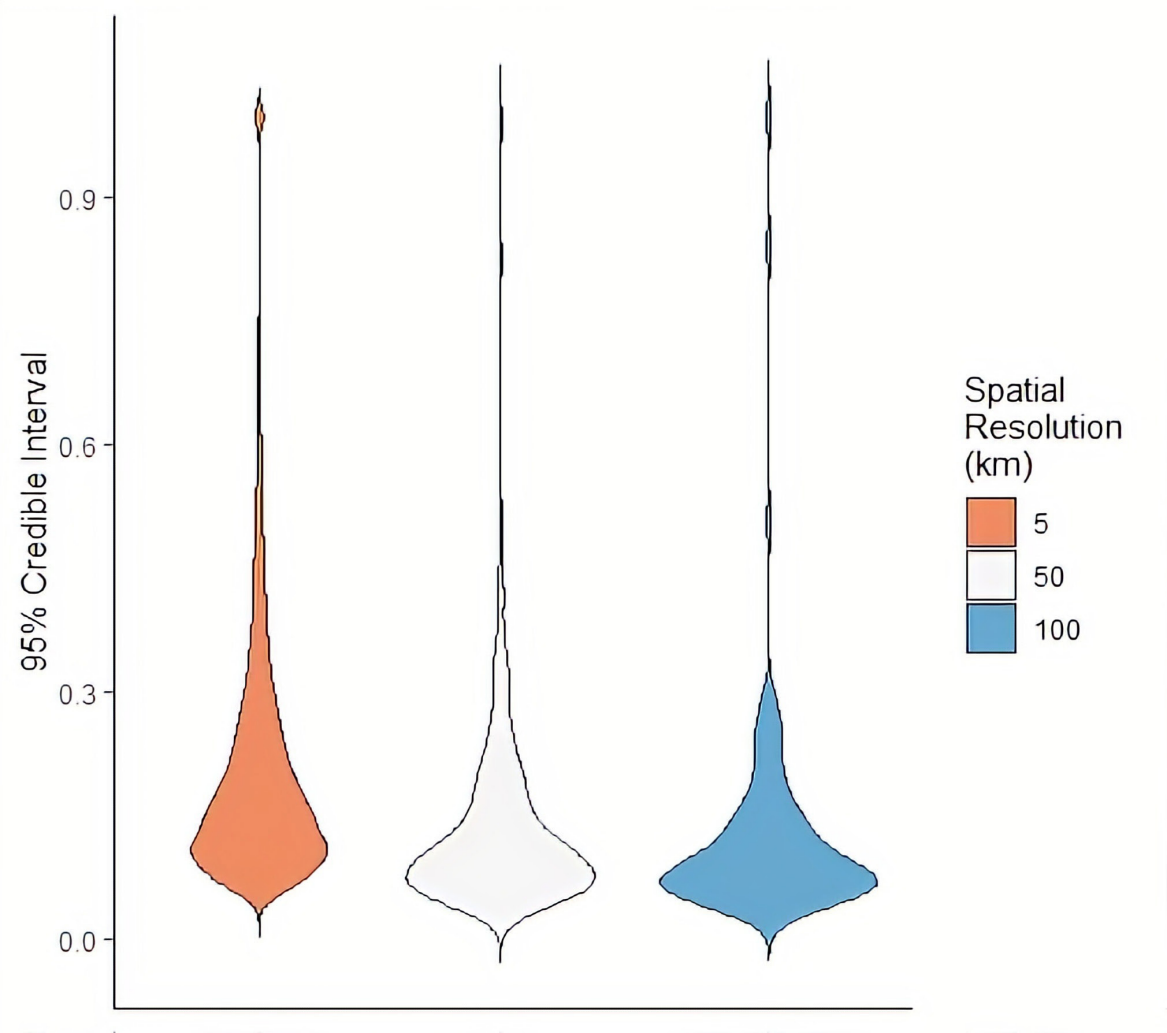
Violin plot of posterior 95% credible intervals for caesarean section estimates predicted at the 5 km, 50 km and 100 km scale.

Figure 3 shows the distributions of the posterior 95% CIs for each model as violin plots. These plots show similar summary statistics as boxplots, while also providing information on probability densities, where thinner sections represent a lower probability of a given value occurring. These estimates approximate the trade-off between spatial resolution and uncertainty, representing the density of the width between the posterior upper and lower 95% CI for each grid cell for the 5 km, 50 km and 100 km surfaces. Here, all models had CI widths ranging from near 0 to 1, but CI width became more narrowly distributed and approached zero with decreasing spatial resolution. Mean density at the 100 km and 50 km scales were 0.13 (±0.14) and 0.14 (±0.13), respectively, while mean density at the 5 km scale was 0.21 (±0.16).

**Figure 4:**
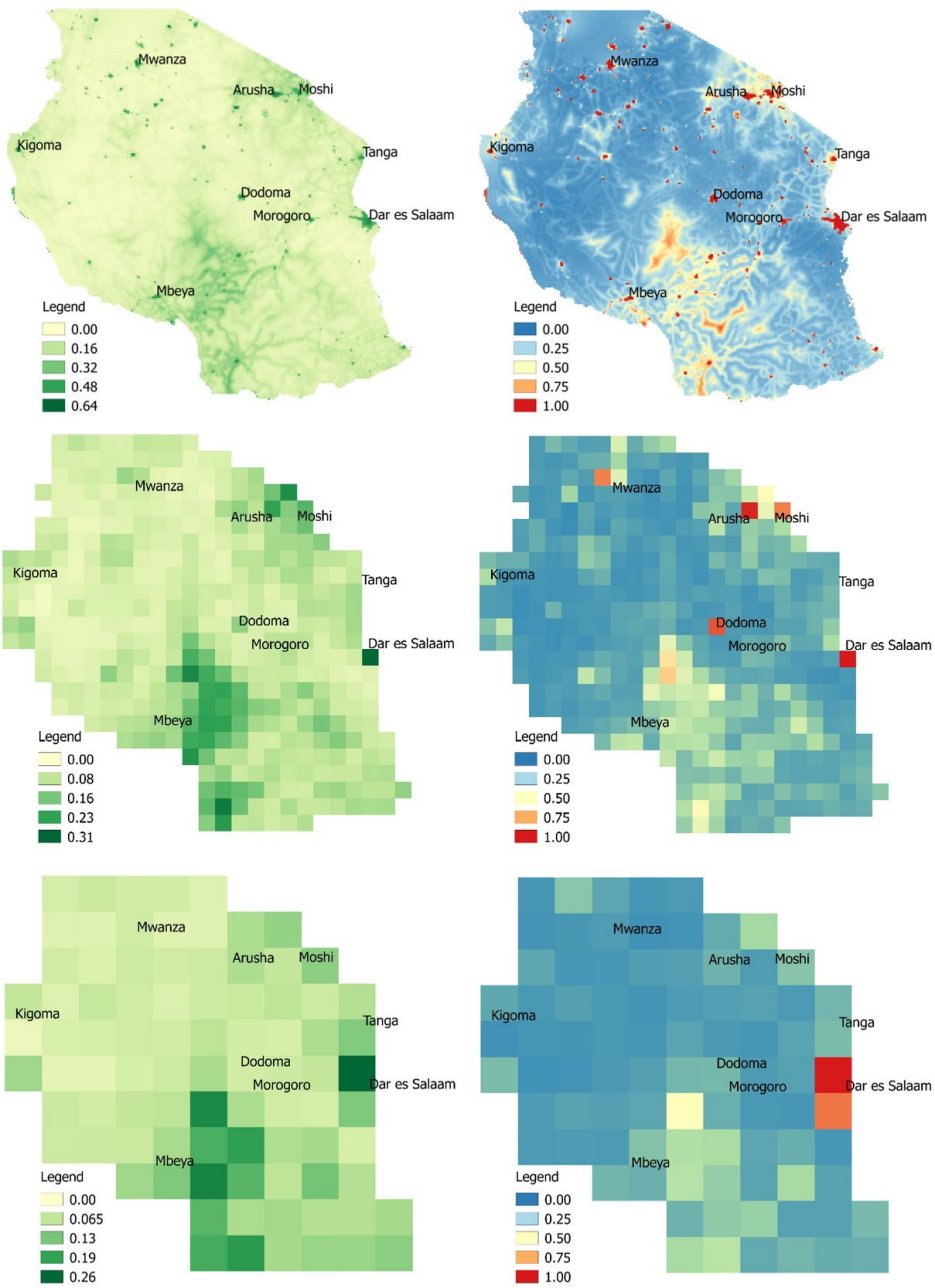
Modelled c-section prevalence estimates (left) and associated 95% credible interval (right) at 5 km (top), 50 km (middle), and 100 km (bottom), Tanzania, 2015.

Figure 4 visualises prevalence of delivery via c-section and associated uncertainty at the 5 km, 50 km and 100 km spatial resolution. These maps show spatial patterns typical of c-section deliveries, with higher prevalence observed in cities such as Arusha and Dar es Salaam, and lower prevalence in areas with high inaccessibility to a health facility or among more impoverished women (table 1). Overall, the mean estimated prevalence of obtaining a c-section at delivery at the 5 km resolution was 8.7% (±6.2%), while the mean uncertainty as measured by the posterior distribution was 20.9% (±15.7%). Mean estimated prevalence was slightly lower at the 50 km and 100 km resolutions, measuring 7.9% (±4.9%) and 7.8% (±4.8%), respectively, while mean uncertainty was 14.2% (±12.7%) and 13.1% (±14.3%). Areas of higher c-section utilisation were associated with higher uncertainty and observed around major urban areas, notably Dar Es Salaam, Arusha and Moshi and Dodoma. This trend was observed across spatial resolutions and in accordance with DHS data. Among DHS regions, Dar Es Salaam had the highest prevalence of delivery via c-section at 17%, compared with a national average of 5.9% (figure 1).

## Discussion

Overall, we found increasing model uncertainty associated with increasing spatial resolution, as quantified by increasing 95% CI widths (figure 3). This is unsurprising, given that increasing spatial resolution comes with problems of increasingly sparse events, zero inflation and missing data.^42^ Furthermore, both c-section prevalence as well as model uncertainty tended to be higher in urban areas, reflecting greater variance within the data. This uncertainty could be due to increased data availability within large population centres, or potentially due to GPS displacement error within urban areas. While Bayesian hierarchical modelling techniques have developed to help account for these limitations through rigorous quantification of uncertainty,^3^ our findings suggest an important and often overlooked trade-off persists between modelling these high-resolution health indicators and corresponding policy relevance if these estimates tend to be highly uncertain. This is evidenced in figure 4, where the highest rates of modelled c-section estimates tend to also have the highest corresponding uncertainty. Despite increasing uncertainty that accompanied increasing spatial resolution, we found that the 5 km model was the most precise, as evidenced by a high precision estimate (table 1). These findings correspond to findings reported in the Tanzania DHS report^23^ where rates of c-section utilisation are higher in urban areas. Other studies similarly suggest that while the prevalence of c-section is increasing globally,^43 44^ women in more rural areas who cannot access a health facility quickly have a lower chance of undergoing the procedure in emergency circumstances.^45 46^

Within our models, we further found that poverty was negatively correlated with undergoing delivery via c-section across spatial resolutions and was significant at lower spatial resolutions (50 km and 100 km). These findings are again in line with reported DHS findings suggesting women in the highest wealth quintile were eight times more likely to undergo a c-section as compared with those in the lowest quintile.^23^ Because health insurance coverage is generally low in Tanzania and relies heavily on payment at point of service,^47^ our findings may suggest that more impoverished women are either unable to afford c-section surgeries when needed or are generally accessing healthcare less frequently across the continuum of pregnancy and childbirth.

Researchers are increasingly quantifying health and development indicators at the district level and high spatial resolutions, with aims of achieving Sustainable Development Goals to ensure ‘no one left behind’.^8 48^ While identifying these previously hidden pockets of vulnerable and marginalised populations is vital to improving the health and well-being of all, the geostatistical methods used to accomplish these goals have inherent uncertainty and bias, which should be communicated effectively to policy makers and other non-academic audiences. While studies have recognised the importance in quantifying this uncertainty,^3 22 42^ no studies have explored how increasing spatial resolution impacts model estimates and uncertainty. This study is therefore the first to map high resolution estimates of c-section prevalence in Tanzania and examine the trade-off between increasing spatial resolution and associated model uncertainty in these estimates. These results of this study imply an important trade-off between identifying concealed spatial heterogeneities and accuracy of estimates, which should be optimally communicated in policy relevant settings.

### Limitations

This methodological study was exploratory in nature, examining the trade-off between increasing spatial resolution and model uncertainty and is therefore subject to a variety of limitations. First, we used a suite of standard covariates to explore the impact of spatial resolution alone on model uncertainty, and therefore rigorous covariate selection and model validation efforts were not undertaken during these analyses. As such, the results of these models may not be generalisable to Tanzania, nor to other study countries, and should be used for illustrative purposes only. Furthermore, the spatial resolutions chosen for these analyses represent a theoretical exploration of the impact of increasing resolution on modelled estimates and are unlikely to represent estimates at resolutions at which policy decisions are made. Future work may explore the impact of increasing spatial resolution on modelled estimates at policy relevant administrative boundaries, as opposed to the rasters employed in these analyses. Second, the suite of covariates and DHS data used are subject to their own biases and limitations—for example, the DHS collects data on births within the previous 5 years, so the estimates presented here may not reflect the current situation within Tanzania. Furthermore, DHS data are not routinely collected registration data and therefore do not capture information on c-sections performed on women who have subsequently died. This potentially represents biased information, as only women obtaining and surviving the procedure are interviewed. Additionally, travel to the nearest public hospital does not account for individuals who may bypass the nearest facility in favour of a facility with higher quality of care. Lastly, the geospatial covariates used have associated error and misclassification bias, particularly night-time lights that may suffer from light refraction errors, for example. Future work should aim to include more recent data on actual health facility used, where possible, and explore the impact of misclassification bias inherent to these environmental covariates.

## Conclusions

Researchers are increasingly applying Bayesian hierarchical modelling techniques to visualise high-resolution spatial patterns of health indicators. These techniques offer powerful and rigorous methods to quantify and visualise model uncertainty, but few studies have explored how to communicate this uncertainty in policy-relevant settings. Here, we explored how model uncertainty changes with increasing spatial resolution and found that while uncertainty increases with higher spatial resolutions, model precision was best approximated at the highest spatial resolution, suggesting an important policy trade-off between identifying concealed spatial heterogeneities in health indicators. In modelling delivery via c-section at multiple spatial resolutions, we demonstrate poverty to be negatively correlated across spatial resolutions, suggesting important disparities in obtaining life-saving obstetric surgery persist across sociodemographic factors. This work is the first study to explore modelled c-section estimates and uncertainty at varying spatial resolutions and has potential policy implications in terms of visualising spatial patterns of obstetric surgery, as well as focusing MNH data collection efforts within Tanzania.
