# Supplementary material for: Estimating uncertainty in geospatial modelling at multiple spatial resolutions: the pattern of delivery via caesarean section in Tanzania
Source: BMJ Glob Health. 2020 Feb 10;4(Suppl 5):e002092. doi: 10.1136/bmjgh-2019-002092 (PMC7044704; doi:10.1136/bmjgh-2019-002092)
Supplement: Supplementary data [file bmjgh-2019-002092supp001.pdf]

SUPPLEMENTAL INFORMATION

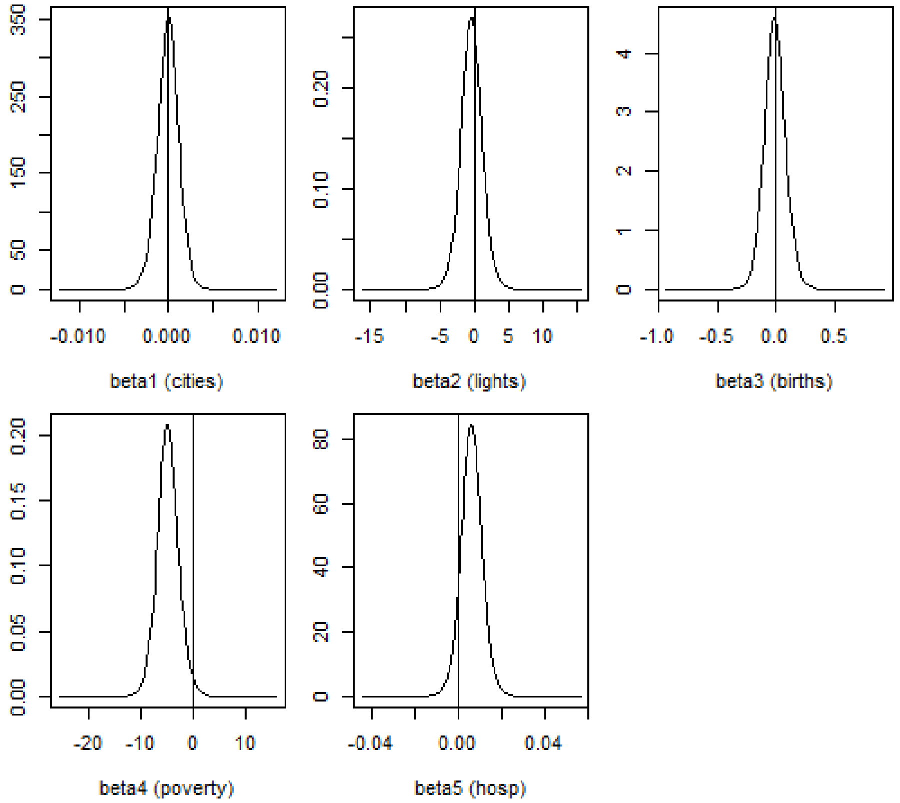

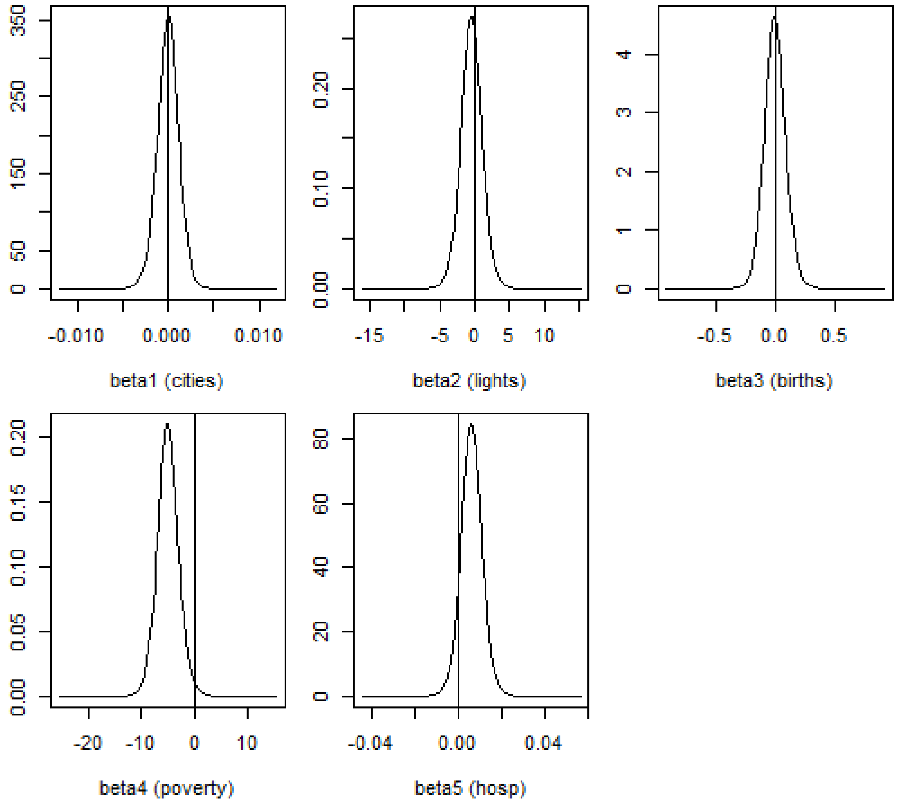

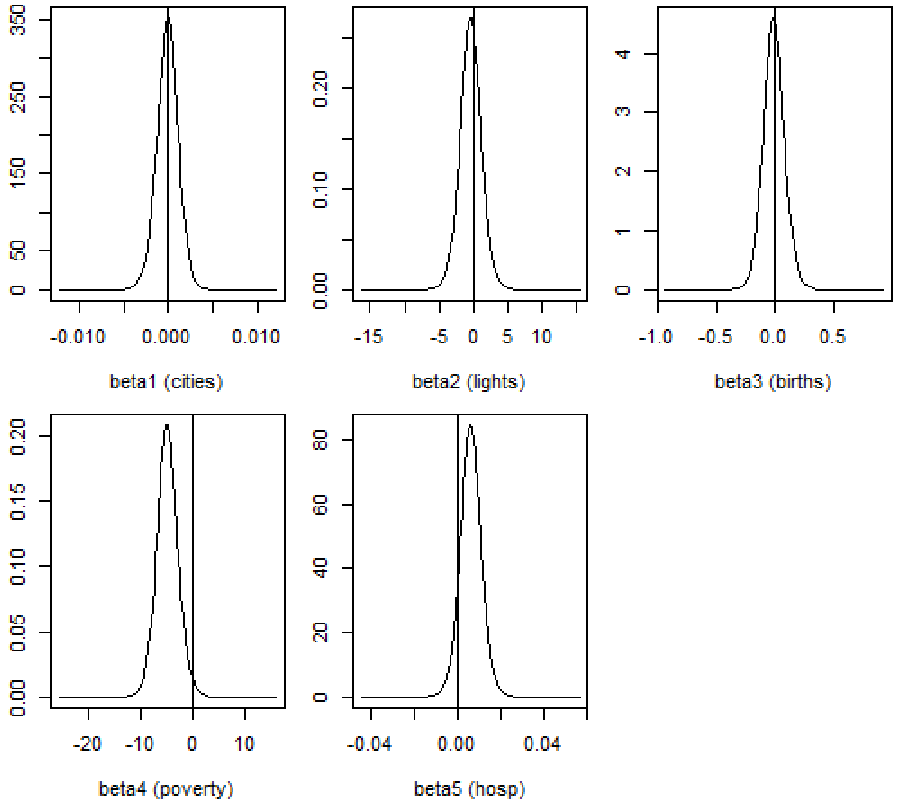

Figure S1. Marginal effect density plots at 5km (top), 50km (middle), and 100km (bottom)

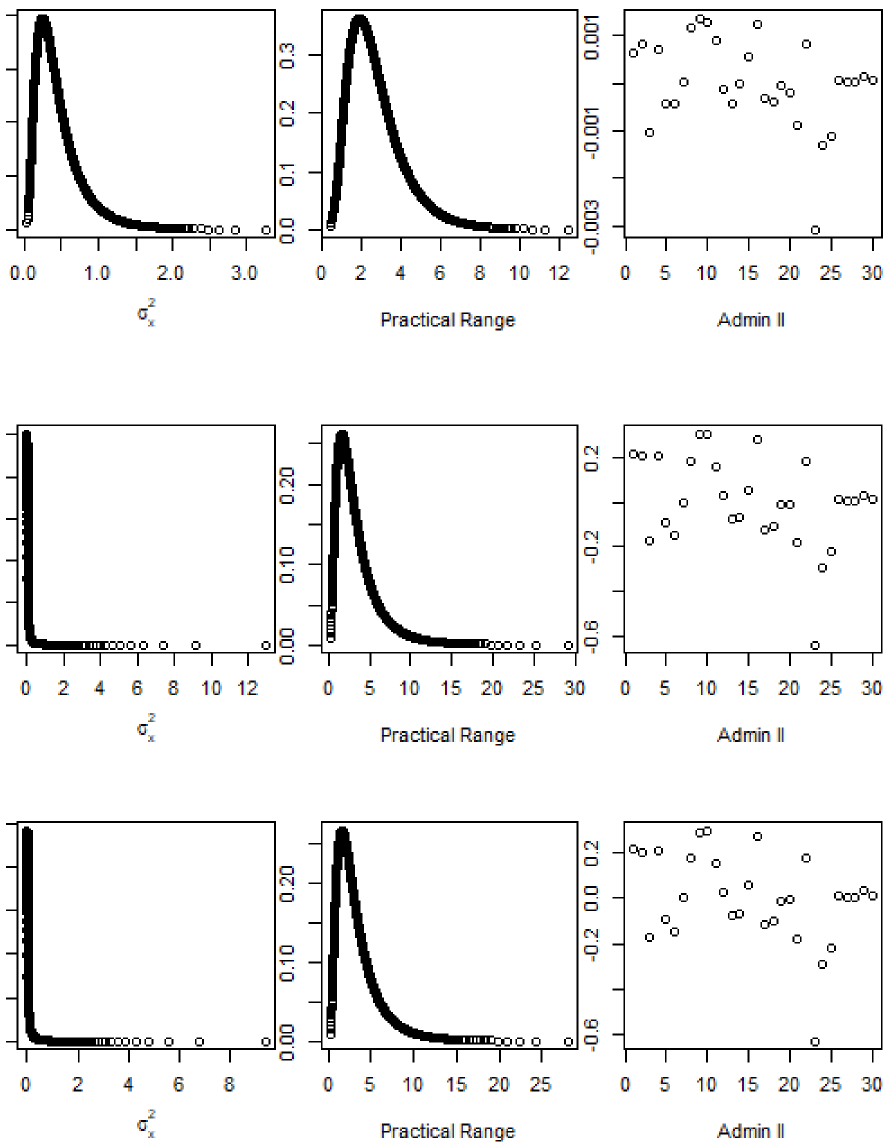

Figure S2. Model variance, model range, and model random effects at the 5km (top), 50km (middle) and 100km (bottom) scales.
